# Oxygen Plasma-Modified Graphene Composite Membranes for Enhanced Forward Osmosis Performance: Mitigating Reverse Salt Flux and Improving Permeability

**DOI:** 10.3390/membranes16030104

**Published:** 2026-03-16

**Authors:** Keyuan Zhang, Yan Wu, Yue Jiang, Qi Han, Minmin Zhang, Li Feng, Liqiu Zhang

**Affiliations:** 1 Beijing Key Laboratory for Source Control Technology of Water Pollution, College of Environmental Science and Engineering, Beijing Forestry University, 35 Qinghua East Road, Haidian District, Beijing 100083, China; 2 Engineering Research Center for Water Pollution Source Control & Ecoremediation, College of Environmental Science and Engineering, Beijing Forestry University, 35 Qinghua East Road, Haidian District, Beijing 100083, China; 3 The Institute of Seawater Desalination and Multipurpose Utilization, Ministry of Natural Resources of the People’s Republic of China, Tianjin 300192, China; 4 Zhejiang Key Laboratory of Petrochemical Environmental Pollution Control, Zhejiang Ocean University, Zhoushan 316022, China

**Keywords:** forward osmosis, monolayer porous graphene, oxygen plasma, separation performance, salt rejection

## Abstract

Forward osmosis (FO) membranes face challenges in balancing high water permeability, low reverse salt flux (RSF), and mechanical durability. Although nanopores in graphene have great theoretical potential, the existing methods make it difficult to independently optimize the nanopores of the graphene layer and the microstructure of the substrate without damaging each other. Here, we propose a defect engineering strategy based on oxygen plasma etching to address this collaborative optimization challenge. Monolayer porous graphene (PG) was integrated with polysulfone (Psf) substrates, followed by oxygen plasma etching to introduce nanopores and oxygen-containing functional groups (e.g., carboxyl, hydroxyl). By controlling the etching time to 10 s, the resulting membrane (S-PG10) exhibited a water flux of 0.24 LMH in 0.5 M NaCl, representing an order-of-magnitude increase compared to the pristine graphene membrane (S-G). Remarkably, S-PG10 maintained a high salt rejection (>96%) and a low *J_s_*/*J_w_* (<0.35 g·L^−1^). Substrate modification via short-term plasma etching (5 min) further doubled the water flux of S*5-PG10 (0.47 LMH in 0.5 M NaCl) by increasing porosity (81.8%→85.6%) and hydrophilicity. However, prolonged etching (>15 min) degraded mechanical strength and increased RSF due to pore structure disruption. To enhance robustness, Poly(D,L-lactic acid) (PDLLA)-doped substrates (S^#^-PG) were engineered, with 0.1 wt.% PDLLA optimizing mechanical properties while maintaining low RSF and high flux. Excessive PDLLA (10 wt.%) induced hydrophobicity and crystalline structures, reducing permeability. The study demonstrates that synergistic optimization of plasma etching duration on the graphene selective layer (5~10 s) and substrates (5 min) as well as PDLLA doping (0.1 wt.%) balances pore architecture, surface chemistry, and substrate integrity, achieving FO membranes with superior water-salt selectivity and mechanical stability. These findings provide critical insights into designing high-performance graphene-based membranes for sustainable desalination and water purification.

## 1. Introduction

Forward osmosis (FO) has emerged as a promising membrane-based separation technology with wide-ranging applications in brine treatment, protein purification, and pharmaceutical processing [1,2]. Unlike conventional thermal methods, FO operates under ambient temperature and pressure, thereby preserving the structural integrity and biological activity of sensitive products such as proteins and drugs [3]. Furthermore, the long-standing challenge of high energy consumption for draw solute regeneration can be alleviated by integrating with renewable energy sources (e.g., solar- or wind-driven regeneration) [4]. Despite these advantages, FO still faces critical challenges, most notably loss of draw solution, increased operational costs, and contamination of the feed solution caused by high reverse salt flux (RSF) [5,6]. Reducing RSF while simultaneously enhancing water permeability remains a central research focus for advancing FO toward practical deployment.

Carbon-based nanomaterials, including carbon nanotubes and graphene, have been extensively explored in membrane development due to their outstanding properties, such as rapid water transport, high mechanical strength, and excellent chemical stability [7,8]. Among them, monolayer porous graphene (PG), a two-dimensional (2D) material, has garnered significant attention due to its capability for ultrafast water transport and nearly complete salt rejection [9]. Monolayer PG was typically obtained by introducing pores into monolayer graphene, an ultra-thin sheet composed of sp^2^-bonded carbon atoms arranged in a hexagonal honeycomb lattice, with a thickness of only 0.34 nm. The inherent impermeability of pristine monolayer graphene, attributed to its narrow geometric gaps (0.064 nm), necessitates the creation of pores through techniques such as plasma oxidative etching or ion bombardment [10]. These processes generate artificial apertures within the graphene structure, facilitating the passage of ions and molecules, thereby achieving the desired monolayer PG configuration.

Building on these fabrication advances, theoretical modeling has also been extensively employed to predict and rationalize the transport behavior of PG membranes. Numerous molecular dynamics (MD) simulations have consistently demonstrated their potential for exceptional water permeability and ion selectivity. [11]. For instance, Konatham et al. investigated the transport behaviors of water and ions through PG pores ranging from 7.5 to 14.5 Å in diameter during RO processes. They found that functionalization of PG with carboxyl groups enhanced ion exclusion, particularly effective in narrow pores even at moderate ionic strengths [12]. Following the theoretical predictions from simulations, several experimental studies have been conducted to explore the practical applications of PG membranes. O’Hern et al. developed polycarbonate track etch (PCTE) membrane-supported PGs featuring tunable sub-nanometer pores achieved through ion bombardment followed by chemical oxidation. They investigated the relationship between pore sizes and PG selectivity using 0.5 M KCl and Allura Red dye as model solutes [13]. Kafiah et al. addressed defects in graphene transferred onto polypropylene (PP) and polyvinylidene fluoride membranes by optimizing interfacial polymerization techniques. They successfully increased the KCl rejection rate of graphene on PP membranes from 57% to 84% [14]. These experimental investigations contribute valuable insights into the development and application of PG membranes across various membrane separation processes, from nanofiltration to selective ion transport, addressing both practical challenges and expanding the potential applications of graphene-based materials in membrane technology.

While previous studies have highlighted the potential of porous graphene (PG) for filtration, most investigations have focused on pressure-driven processes such as reverse osmosis (RO) and ultrafiltration (UF). Several pioneering studies have demonstrated the feasibility of using single-layer graphene membranes in forward osmosis (FO). For example, CVD-grown graphene sheets perforated by O_2_ plasma etching exhibited nearly complete salt rejection in FO desalination tests [15]. However, these studies mainly served as proof-of-concept demonstrations. Systematic understanding of transport behavior remains limited, and the coupling between graphene nanopore selectivity and substrate mass-transfer resistance has not been fully investigated. Recent theoretical and modeling studies provide important insight into this limitation. Forward osmosis differs fundamentally from pressure-driven desalination because the driving force is an osmotic gradient rather than hydraulic pressure. Nonequilibrium molecular dynamics simulations have shown that ions confined within membrane nanochannels can accumulate and increase hydraulic resistance to water transport, thereby altering effective selectivity [16]. In addition, transport analyses indicate that performance loss in FO membranes largely originates from diffusion limitations inside the porous support layer, leading to internal concentration polarization (ICP), which reduces the effective osmotic driving force [17]. These findings suggest that FO performance is governed by coupled mass transfer through both the selective layer and the support structure, rather than by graphene nanopore sieving alone. To fully realize the advantages of PG in FO, it is not only necessary to optimize the PG-based active layer but also to address the structural characteristics of the support layer, which play a pivotal role in governing water flux, ICP, and overall separation performance [18]. Polysulfone (Psf), a widely used support material, suffers from relatively poor hydrophilicity, which limits water permeability. Several strategies have been developed to improve Psf substrates, including blending with hydrophilic additives (e.g., cellulose esters, sulfonated polysulfone) and surface carboxylation [19,20]. Similar behavior has been reported in conventional thin-film composite (TFC) FO membranes, where incorporation of hydrophilic SiO_2_ into the porous substrate can markedly enhance water flux by alleviating ICP [21]. Oxygen plasma treatment offers a particularly attractive route, as it can simultaneously introduce hydrophilic functional groups to enhance wettability and engineer surface defects to tailor pore structures [22].

In this study, three novel membranes (S-PG, S*-PG and S^#^-PG membranes) were prepared by oxygen plasma etching of monolayer PG and Psf-based substrates, respectively. These membranes were then applied in a laboratory-scale FO device to investigate their desalination efficiency in a 0.5 M draw solution (DS). The research aimed to elucidate the practical separation behavior of the PG and substrate in FO by combining membrane performance evaluation with comprehensive characterization, including morphology, wettability, and surface chemistry analysis. The findings were crucial for advancing the understanding of PG’s selectivity in FO applications and provide insights for further development and optimization of graphene-based membranes for efficient seawater desalination processes.

## 2. Materials and Methods

### 2.1. Materials and Chemicals

Polysulfone beads (Psf, Mn: 220,000 Da, Sigma-Aldrich, St. Louis, MO, USA), polyethylene glycol (PEG-400, Mw: 400 Da, analytical reagent, Sinopharm Chemical Reagent, Shanghai, China), 1-methyl-2-pyrrolidinone (NMP, anhydrous, 99.5%, Sigma-Aldrich, St. Louis, MO, USA) and N, N-dimethylformamide (DMF, anhydrous, 99.8%, Sigma-Aldrich, St. Louis, MO, USA) were used to prepare the polymer substrate. Pristine monolayer graphene (25 μm thick) grown on copper foils by chemical vapor deposition (CVD) was purchased from 6Carbon Technology, Shenzhen, China. Ferric chloride (FeCl_3_, analytical reagent, Beijing Chemical Work, Beijing, China) was dissolved in deionized (DI) water to prepare a copper etchant, while anhydrous ethanol (J. T. Baker (Avantor), Phillipsburg, NJ, USA) was used to remove residual etchant during the graphene transfer process. Poly(D,L-lactic acid) (PDLLA) (Mw: 60 kDa) purchased from Jinan Daigang Biomaterial Co., Ltd., Jinan, China, was employed to enhance the mechanical strength of the Psf substrate.

Potassium chloride (KCl), sodium chloride (NaCl) and magnesium chloride hexahydrate (MgCl_2_∙6H_2_O), all analytical reagents from Sinopharm Chemical Reagent, Shanghai, China, were used to prepare DSs for the FO desalination process.

### 2.2. Preparation and Modification of Polymer Substrates

a. Fabrication of Polymer Substrates (S): The polymer substrates were prepared using non-solvent-induced phase separation. A homogeneous casting solution was obtained by stirring a mixture of Psf (12 g), PEG-400 (6 g), DMF (20.5 g) and NMP (61.5 g) for 10 h, followed by overnight degassing. The solution was then cast onto a clean glass plate using an adjustable film applicator (3530, Elcometer, Manchester, UK) set at a gate height of 100 μm. The cast film was immediately immersed in a DI water bath for 10 min until the substrate formed. After soaking in DI water for 24 h to remove residual solvents, the substrate was thoroughly dried and stored in a desiccator for future use.

b. Modification of Psf substrates (S*): To investigate the effect of oxygen plasma etching time on the structural properties of Psf substrates, a plasma cleaner (P3C, Schwarze, Althengstett, Germany) was used. The operating conditions included pure oxygen injection, a gas flow rate of 320 sccm, power of 100 W, vacuum pressure of 0.012 kPa, and etching times of 5 min, 15 min and 30 min. The modified substrates were labeled as S*5, S*15 and S*30, respectively, based on the plasma treatment duration.

c. Preparation of mechanically reinforced Psf substrates (S^#^): The mechanically reinforced PDLLA/PSf blended substrates were prepared as follows: PSf and PDLLA were dissolved in a mixed solvent of NMP and DMF, with PDLLA mass ratios of 0.1%, 1%, 10%, corresponding to the labels S^#^/P0.1, S^#^/P1, and S^#^/P10, respectively (Table S1). The solution was continuously stirred to achieve a homogeneous casting solution. A 100 µm thick liquid film was then cast using a film applicator, and the substrates were fabricated via the phase inversion method outlined in part (a) of Section 2.2. The resulting substrates were stored in deionized water at 4 °C for future use.

### 2.3. Preparation of S-PG, S*-PG and S^#^-PG Composite FO Membranes

**Figure 1:**
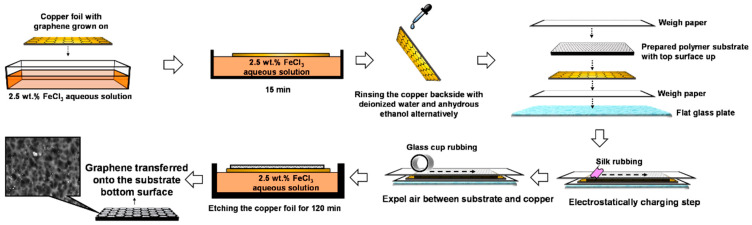
The transfer procedure of CVD monolayer graphene from copper to the bottom surface of the prepared polymer substrate.

Figure 1 illustrates the graphene transfer procedure used in this study. First, the backside of the copper foil was treated with a 2.5 wt.% FeCl_3_ aqueous solution for 15 min to remove graphene flakes grown on the back. The foil was then alternately rinsed with ethanol and deionized (DI) water. Next, the copper foil was placed graphene-side up on a piece of weighing paper atop a glass plate. The dried polymer substrate was positioned with its bottom side in contact with the graphene. A second piece of weighing paper was placed on top, followed by rubbing with a polyester mesh to ensure firm adhesion between the substrate and the copper foil through static electricity. The stack was gently pressed with a cup wall to expel any trapped air. Finally, the copper foil, with the substrate adhered, was floated on a 2.5 wt.% FeCl_3_ aqueous solution for 90–120 min until the copper was completely etched. This process produced the substrate-supported graphene (S-G). After thoroughly rinsing off residual etchant with DI water, the S-G was air-dried at 60 °C for 12 h and stored in a desiccator for later use.

Oxygen plasma treatment can introduce structural defects (i.e., permeable pores) into the graphene crystal lattice and incorporate oxygen-containing functional groups onto the graphene surface or defect edges. However, excessive plasma treatment may compromise the ion selectivity of porous graphene (PG). To achieve high-performance PG membranes, the prepared S-G membranes were treated with oxygen plasma for 10 s, 20 s, and 30 s, resulting in S-PG10, S-PG20, and S-PG30, respectively (Table S2). The treatment was conducted using a plasma cleaner (P3C, Schwarze, Althengstett, Germany) operating at 40 W radio frequency power with a pure oxygen flow under a pressure of 12 Pa.

The optimal oxygen plasma treatment duration for porous graphene, determined through FO performance with S-PG membranes, was selected for the further preparation of S*-PG and S^#^-PG membranes. The preparation of S*-PG membranes followed the same procedure as for S-PG membranes, except that the substrates used were S*, modified by oxygen plasma. The resulting membranes were designated as S*5-PG, S*15-PG, and S*30-PG (Table S3), corresponding to substrate etching durations of 5 s, 15 s, and 30 s, respectively. Similarly, S^#^-PG membranes were prepared from PDLLA/PSf blended dope for enhanced mechanical strength and subsequently modified by oxygen plasma with the optimal etching duration determined from the FO tests using S*-PG membranes. The resulting membranes were labeled as S^#^/P0.1-PG, S^#^/P1-PG and S^#^/P10-PG, corresponding to PDLLA mass ratios of 0.1%, 1%, 10%, respectively.

### 2.4. Membrane Characterizations

To minimize interference from water, all membrane samples were thoroughly dried prior to characterization. The thickness of the S-PG membranes was measured using a digital micrometer (series 293-240, Mitutoyo, Kawasaki, Japan). Measurements were taken at eight different locations, and the average value was reported.

The mechanical strengths of the S, S-G, S-PG, S*, and S^#^ were measured using an electronic universal material test machine (WDW3020, Ke Xin Experimental Instrument Research Institute, Changchun, China) under a tensile rate of 2 mm/min.

The wettability of the substrate, S-G and all S-PG, S*-PG, S^#^-PG membranes was determined through static contact angle (CA) measurements using a CA goniometer (OCA20, Dataphysics, Filderstadt, Germany). A 2 μL drop of DI water was dispensed on the membrane surface, and ten CA measurements were conducted for each membrane type, with the results averaged.

Substrate porosity was assessed using the dry-wet weight method. An analytical balance (ML104, Mettler Toledo, Zurich, Switzerland) was applied to measure the substrate mass before and after complete wetting, and the porosity (*ε*) was calculated using the following equation:

(1) $$\varepsilon (\%)=\frac{(m_{wet}-m_{dry})/\rho_{w}}{{(m_{wet}-m_{dry})/\rho }_{w}+m_{dry}/\rho_{p}}\times 100$$

where *m_wet_* is the mass of the wet sample, g; *m_dry_* is the mass of the dry sample, g; *ρ_w_* is the water density, g/cm^3^; and *ρ_p_* is the polysulfone density, g/cm^3^. Each sample was measured six times and the average results were reported.

The morphologies of the S, S*, S^#^ and S-G membranes were examined using scanning electron microscopy (SEM, Zeiss Merlin, Jena, Germany) without additional material sputtering. A Raman spectrometer (950 FT-Raman spectrometer, Thermo Nicolet, Madison, WI, USA) equipped with a 532 nm laser source (2.33 eV) was employed for Raman analysis. Fourier-transform infrared spectroscopy (FTIR, Bruker, Ettlingen, Germany) was performed to analyze the substrates and membranes, with a spectral scan range of 400–4000 cm^−1^. X-ray photoelectron spectroscopy (XPS, PHI Quantera SXMX, ULVAC-PHI, Chigasaki, Japan) equipped with a focused monochromatized X-ray source (Al Kα = 55 eV) was used to investigate the surface chemical composition, elemental states, and chemical characteristics of the substrates and fabricated FO membranes.

### 2.5. Separation Performance Measurements

#### 2.5.1. Intrinsic Transport Properties of the Substrate and FO Membranes

A dead-end stainless steel cell (XFUF07601, Millipore Corporation, Billerica, MA, USA) was employed to evaluate the intrinsic transport properties of the Psf substrate and the fabricated FO membranes. The measurements included the pure water permeability (PWP) of the substrate, along with the water permeability (A), salt permeability (B), and salt rejection (R) of the fabricated FO membranes, all assessed under reverse osmosis (RO) mode. The effective membrane area for these measurements was 40 cm^2^. The PWP value of the substrate was determined by calculating the pure water flux under a transmembrane pressure of 0.2 MPa, as described by Equation (2).

(2) $$PWP=\frac{M}{\rho_{w}\cdot S_{m}\cdot ∆t}$$

where *M* represents the mass of the filtrate, g; Δ*t* is the filtration time, h; *ρ*_w_ denotes the water density, g/cm^3^; and *S_m_* is the effective membrane area, cm^2^.

The water permeability coefficients (A), salt rejections (R) and salt permeability coefficients (B) of the fabricated FO membranes were also tested using the dead-end cell. The specific testing procedure was as follows: a 50 mM NaCl solution was prepared as the feed solution and filtered under a pressure of 0.35 MPa to measure the permeate water flux and salt rejection rate of the FO membranes. The conductivity of the filtrate was measured using a conductivity meter, and the salt concentration in the solution was calculated based on the relationship between conductivity and concentration. The values of A (LMH/bar) and R (%) can be calculated from Equations (3) and (4), while B (LMH) was calculated according to Equation (5), derived from the solution-diffusion theory [23]:

(3) $$A=\frac{V_{p}}{t\cdot S_{m}}$$

(4) $$R=\frac{{1-C}_{p}}{C_{0}}\times 100$$

(5) $$B=A\frac{1-R}{R}(∆P-∆\pi )$$

where *V_p_* is the permeate volume, L; *C_p_* is the salt concentration in the permeate, mol/L; *C*_0_ is the salt concentration in feed solution, mol/L; Δ*P* is the applied hydraulic pressure and Δ*π* is the osmotic pressure across the membrane, bar.

#### 2.5.2. FO Performance of the S-PG, S*-PG and S^#^-PG Membranes

A house-made lab-scale FO setup was employed to evaluate the water flux and reverse salt flux (RSF) of the S-G and all S-PG membranes. The schematic diagram of the FO setup is shown in Figure S1. The experiments were conducted using a membrane with an effective separation area of 3 cm^2^, oriented with the graphene or PG side (active layer) facing the feed solution (AL-FS). DI water was used as the FS, while 0.5 M solutions of KCl, NaCl, and MgCl_2_ served as the draw solutions (DSs). The FO tests were carried out at room temperature for a duration of 24 h, with the flow velocity of both the FS and DS maintained at 2.0 cm/s across the membrane surface.

The water flux (*J_w_*, LMH) was measured by recording the mass increase in DS using an analytical balance (ML104, Mettler Toledo, Zurich, Switzerland) and calculated using Equation (6):

(6) $$J_{w}=\frac{∆m_{D}}{\rho_{w}\cdot S_{m}\cdot ∆t_{w}}$$

here, Δ*m_D_* represents the mass increase in DS, g; and Δ*t_w_* is the time interval during which the DS mass increased, h.

The RSF (*J_s_*, gMH)) was determined by measuring the salt concentration in the FS using a conductivity meter (DDSJ-308F, Rex, Shanghai, China) and calculated by Equation (7):

(7) $$J_{s}=\frac{m_{s}}{S_{m}\cdot ∆t_{s}}$$

here, *m_s_* represents the final salt mass in FS, g; Δ*t_s_* is the recorded time interval during which the salt reversely transported, s. The specific salt flux (*J_s_*/*J_w_*, g/L) is defined as the ratio of RSF to water flux.

## 3. Results and Discussion

### 3.1. Structural Regulation and Mass Transfer Behavior of S-PG Membranes

#### 3.1.1. Physiochemical Properties of Graphene Deposited Membranes

**Figure 2:**
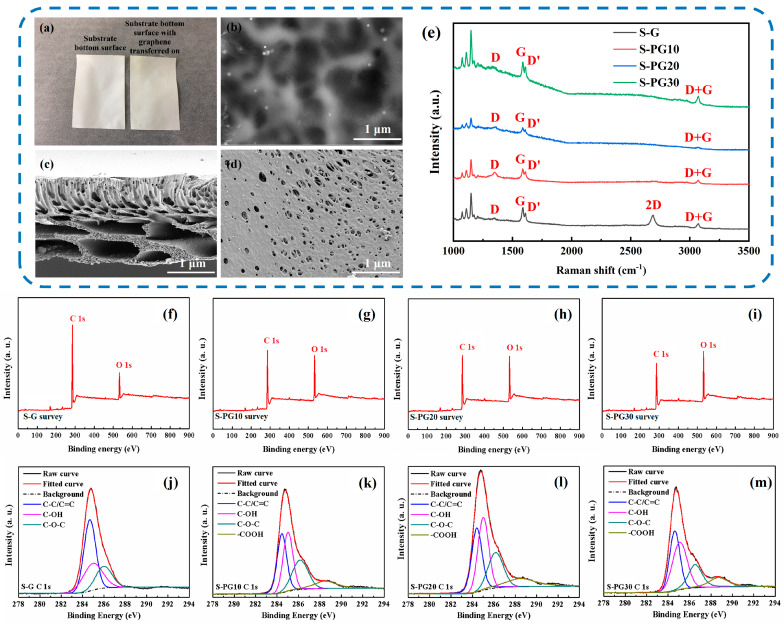
Comparison images of the bare substrate and the substrate with deposited graphene (**a**); SEM image of the graphene-deposited surface (**b**); SEM image of the cross section (**c**) and the bottom surface of the Psf substrate (**d**); Raman spectra of S-G and S-PG membranes (**e**); XPS survey spectra (**f**–**i**) and the C 1s XPS spectra (**j**–**m**) of S-G and S-PG membranes.

The successful transfer of a continuous monolayer graphene onto the polysulfone (Psf) substrate is confirmed by the distinct color change (Figure 2a) and the presence of characteristic wrinkles on the surface (Figure 2b). The Psf substrate exhibits a typical asymmetric three-layer structure (Figure 2c,d): a dense top layer, a finger-like porous middle layer, and a macrovoid-containing bottom layer, consistent with previously reported Psf FO supports [23].

To tailor the membranes for FO, the graphene layer in the S-G membrane was selectively modified via oxygen plasma etching to create a series of nanoporous graphene (S-PG) membranes. Raman spectroscopy was employed to evaluate the structural disorder of graphene. Raman spectroscopy (Figure 2e) confirms the structural evolution: both the S-G and S-PG membranes exhibit the characteristic D (1350 cm^−1^), G peak (1586 cm^−1^), and 2D peak (2688 cm^−1^) bands of graphene. Compared with S-G, the plasma-etched S-PG membranes show a pronounced increase in the D-band intensity, indicating the introduction of structural defects. Since the D band originates from defect-activated breathing modes of sp^2^ carbon rings, and the G band corresponds to the in-plane vibration of ordered sp^2^ carbon atoms in a lattice, the *I_D_*/*I_G_* ratio serves as a reliable semi-quantitative indicator of defect density. The *I_D_*/*I_G_* ratio increases markedly from 0.19 (S-G) to 0.92 (S-PG10), suggesting a substantial increase in defect sites after plasma treatment. Such vacancy-type defects, together with edge oxidation, may correspond to the formation of defect openings that function as selective transport pathways within the graphene lattice [10].

X-ray photoelectron spectroscopy (XPS) further confirms the plasma-induced chemical modification of the graphene surface. The XPS survey spectra (Figure 2f–i) show that the C/O ratio decreases with increasing oxygen plasma treatment duration, indicating effective carbon etching and the incorporation of oxygen-containing functional groups. Combined with the increase in the Raman *I_D_*/*I_G_* ratio, these results together suggest the generation of defect sites that can act as selective transport pathways. The C1s spectra (Figure 2j–m) identify three primary carbon bonds: (1) C-C/C=C (sp^2^/sp^3^ hybrid bonds), (2) C-O (hydroxyl groups), and (3) C-O (epoxy groups), which are present in both the S-G and S-PG membranes. Notably, carboxyl groups (C=O) emerged in S-PG membranes. A distinct chemical shift from C-O-C/C-OH to C=O/COOH groups is observed with increased etching time (Figure S2), directly correlating with the enhanced surface hydrophilicity discussed below.

#### 3.1.2. Wettability and Mechanical Robustness of Psf Substrate, S-G and S-PG Membranes

**Figure 3:**
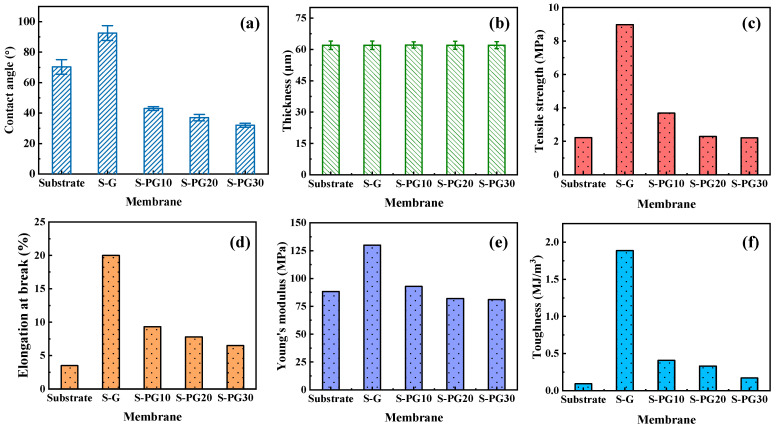
Contact angles (**a**), thickness (**b**), tensile strength (**c**), elongation at break (**d**), Young’s modulus (**e**) and toughness (**f**) of the Psf substrate, S-G and S-PG membrane surfaces.

Surface wettability, a critical factor for membrane performance, is significantly altered by plasma etching (Figure 3a). The S-G membrane is hydrophobic (CA = 92 ± 4.9°), while all S-PG membranes become hydrophilic (CA < 45°), with CA decreasing progressively with etching time. This enhancement was attributed to the increased density of polar oxygen-containing functional groups, as evidenced by XPS (Figure 2f–m) [24].

Mechanical properties are crucial for FO operational stability. The composite S-PG membranes maintain a consistent thickness of ~62 μm (Figure 3b). As shown in Figure 3c–f, the S-G membrane, reinforced by the pristine graphene layer, exhibits superior tensile strength, elongation at break, Young’s modulus, and toughness compared to the bare Psf substrate. After plasma etching, the mechanical properties of S-PG membranes decrease, with S-PG30 showing values close to the Psf substrate. This trend confirms that while the graphene layer enhances mechanical strength, the introduction of nanopores compromises the structural integrity of the 2D lattice. Importantly, S-PG10 retains significantly better mechanical properties than the Psf substrate, indicating a balance between porosity and robustness [25].

#### 3.1.3. FO Performances of S-G and S-PG Membranes

**Figure 4:**
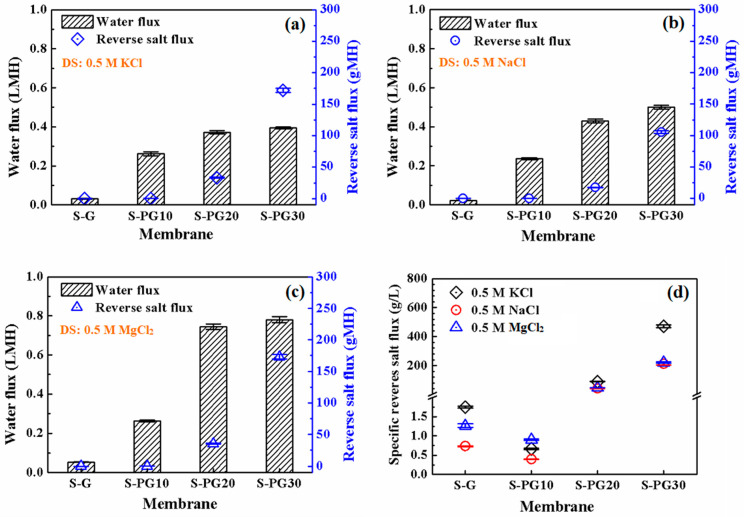
Water fluxes and RFSs of the S-G and S-PG membranes in FO using 0.5 M KCl (**a**), 0.5 M NaCl (**b**) and 0.5 M MgCl_2_ (**c**) as the draw solutions; and the specific reverse salt flux (**d**) of the S-G and S-PG membranes under each draw solution.

The FO performance of S-G and S-PG membranes was evaluated in terms of water flux (*J_w_*), RSF (*J_s_*), and specific salt flux (*J_s_*/*J_w_*) using different draw solutions (DSs) (0.5 M KCl, NaCl, and MgCl_2_) (Figure 4). A consistent trend is observed across all DSs (Figure 4a–c): Plasma etching significantly alters the transmission characteristics, increasing *J_w_*, but simultaneously causing an increase in *J_s_*.

The S-PG10 membrane demonstrates optimal performance. Its *J_w_* (e.g., 0.26 LMH for KCl) is 4–10 times higher than that of S-G membrane, while its *J*_s_ increases only 2–5 times. Consequently, the *J*_s_/*J*_w_ ratio (Figure 4d), a key indicator of membrane selectivity, reaches its lowest value for S-PG10. This is because an etching duration of 10 s created high-density nanoporous defects, as evidenced by the increased *I_D_*/*I_G_* ratio in Figure 2e. In addition to the artificial pores, the hydrophilic deprotonated hydroxyl (CO^−^) and carboxylate (COO^−^) groups introduced at the pore edges via oxygen plasma etching enhance both interfacial hydration and electrostatic repulsion toward ions [21]. Thus, although water transport is significantly promoted, ion permeation does not increase proportionally in *J_s_*. An optimal balance between permeability and selectivity is therefore achieved. For S-PG30, the sharp increase in the observed *J_s_* value is due to excessive etching, which leads to excessive pore defects and thereby damages the structural integrity of graphene, as evidenced by the decline in mechanical properties.

Regarding RSF, S-PG10 exhibits RSFs of 0.17, 0.09, and 0.24 gMH for 0.5 M KCl, NaCl, and MgCl_2_, respectively, which were 2, 5, and 3 times higher than those of S-G. While the difference in water flux between S-PG10 and S-G was more pronounced, the difference in RSF values was relatively smaller. After converting the RSF values to molar mass units (KCl: 74.55 g/mol; NaCl: 58.44 g/mol; MgCl_2_: 95.21 g/mol), the reverse salt flux for KCl, NaCl, and MgCl_2_ in the feed solution was calculated to be 2.28, 1.54, and 2.52 mmol/(m^2^·h), respectively. The RSF values followed the order of RSF(MgCl_2_) > RSF(KCl) > RSF(NaCl) after 24 h of FO desalination. This trend could be attributed to the greater driving force for the solvent molecules passing through the membrane resulting from the higher osmotic pressure of MgCl_2_ (1.12 × 10^−3^ bar) compared to KCl (6.18 × 10^−4^ bar) and NaCl (8.25 × 10^−4^ bar) at the same concentration of 0.5 M [24]. Additionally, the negatively charged hydroxyl and carboxyl groups at the pore edges of S-PG10 membrane likely attracted cations, with Mg^2+^ having a significantly higher charge density than Na^+^ and K^+^, which potentially influenced the salt transport dynamics. Therefore, although the osmotic pressures of NaCl and KCl are similar, the smaller hydrated radius of K^+^ (~0.315 nm) compared to Na^+^ (~0.36 nm) facilitates its easier transport through the membrane pores, resulting in a higher RSF for KCl. The changes in the ratio of *J*_s_/*J*_w_ of S-G and S-PG membranes under different DSs in Figure 4d prove this.

Furthermore, Table S4 summarizes the FO draw solution rejection (R%) values. Despite the defects introduced by oxygen plasma etching, all S-PG membranes maintain exceptionally high salt rejection rates (R > 96%).

### 3.2. Characterizations and Forward Osmosis Performances of S*-PG Membranes

The previous section demonstrated that the fabricated S-PG membranes, particularly S-PG10, exhibited significantly enhanced water flux, excellent permselectivity, and high salt rejection, establishing their potential for forward osmosis (FO) desalination applications. These improvements were attributed to the tailored pore structure, hydrophilic surface modifications, and ion-selective transport behavior introduced by oxygen plasma modification. To further reduce ICP and optimize FO performance, oxygen plasma etching was applied to the Psf substrate for varying durations, while maintaining a fixed etching duration of 10 s for the graphene layer. The physiochemical properties and FO performance of the resulting S*-PG membranes were systematically evaluated to identify the optimal oxygen plasma modification conditions.

#### 3.2.1. Physiochemical Properties of S* Substrates and S*-PG10 Membranes

**Figure 5:**
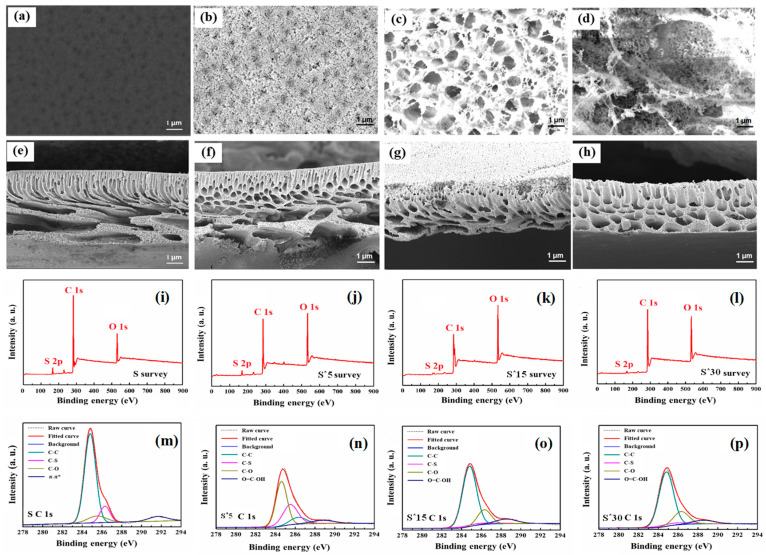
SEM images of the upper surfaces (**a**–**d**) and cross sections (**e**–**h**) of S* substrates; XPS survey spectra (**i**–**l**) and the C 1s XPS spectra (**m**–**p**) of S* substrates.

The SEM images in Figure 5a–h illustrate the upper surface and cross-sectional morphology of the substrates modified by oxygen plasma etching (S*). As the plasma etching duration increased, the surface pore diameter of the S* substrates progressively enlarged (Figure 5a–d), and the dense layer became thinner (Figure 5e–h). Notably, after 30 min of etching, the S*30 substrate exhibited excessive etching, leading to the disruption of the epidermal structure and exposure of the finger-like sublayer beneath the dense layer. While excessive plasma etching of the Psf substrate enhanced water permeability by compromising the dense layer, the resulting larger and overly loose pore structure negatively impacted the separation performance of the graphene (PG) active layer.

The impact of oxygen plasma etching duration on substrate structure was analyzed using FTIR. As shown in Figure S3, the infrared spectra of the modified S* substrates revealed multiple identical absorption peaks compared to the unmodified Psf substrate. These peaks included the O-H stretching vibration of carboxyl groups (2500–3550 cm^−1^), the coupling peak of dimer O-H in-plane bending and C-O stretching vibrations (1400–1428 cm^−1^ and 1250 cm^−1^, respectively), the dimer O-H out-of-plane rocking vibration (920 cm^−1^), and the C=O stretching vibration (1750–1770 cm^−1^) [10].

Changes in surface composition and chemical states of the S* substrates before and after plasma etching were further characterized using XPS and peak fitting analysis. The XPS survey spectra and C 1s peak fitting results are presented in Figure 5i–p. The surfaces of the S* substrates contained C, O, and S elements (Figure 5i–l), consistent with the Psf material. Notably, the oxygen content on the surfaces of S*5, S*15, and S*30 (25.25%, 24.44%, and 20.79%, respectively) was significantly higher than that of the unmodified S substrate (12.28%) (Table S5). The surface O/C ratio also increased after etching, reflecting enhanced oxygen incorporation. As shown in Figure 5m–p, the C 1s spectrum of the unmodified Psf substrate displayed three characteristic peaks at 284.8 eV, 285.5 eV, and 286.3 eV, corresponding to the C-C, C-S, and C-O bonds in Psf, respectively. After plasma modification, a new prominent peak appeared at 288.5 eV, attributed to the C=O bond in carboxyl groups. This new peak resulted from the surface oxidation reaction of the Psf material during oxygen plasma exposure. The process generated significant free radicals, which initiated chain reactions and led to the formation of oxygen-containing functional groups, including carboxyl groups, on the substrate surface [26].

#### 3.2.2. Wettability and Mechanical Robustness of S* Substrates and S*-PG10 Membranes

**Figure 6:**
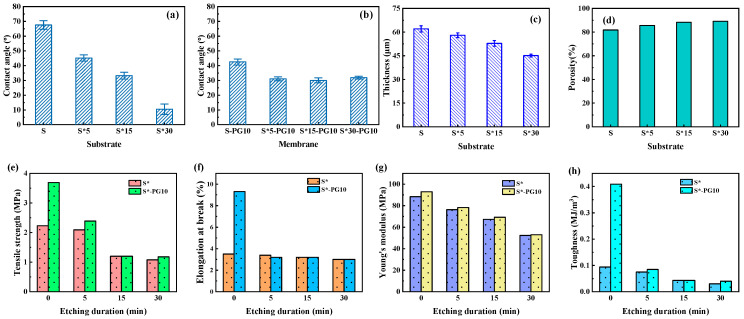
Contact angles (**a**,**b**) of the S* substrate and S*-PG10 membrane; thickness (**c**) and porosity (**d**) of the S* substrate; tensile strength (**e**), elongation at break (**f**), Young’s modulus (**g**) and toughness (**h**) of the S* substrate and S*-PG10 membrane.

Figure 6a,b illustrates the static contact angles of plasma-etched S* substrates and the corresponding S*-PG10 FO membranes. As plasma etching time increased, the hydrophilicity of the S* substrates improved significantly, with the contact angle decreasing from 67.5° for the unmodified S substrate to 10° for the S*30 substrate (Figure 6a). This enhancement was attributed to the formation of polar hydrophilic groups on the substrate surface and an increase in surface porosity (Figure 5a–h).

The S*-PG10 composite FO membranes were fabricated using S* substrates and PG10, and their contact angles were measured to evaluate hydrophilicity. As shown in Figure 6b, the contact angle of the S*-PG10 membranes was approximately 30°, lower than that of the unmodified S-PG10 membrane (45°). Interestingly, the contact angles of the S*-PG10 membranes remained consistent at around 30°, regardless of further increases in plasma etching time for the Psf substrate. This stability suggests that the wettability of the substrate was modulated by the overlying porous graphene layer. The consistent contact angle is likely due to the influence of the van der Waals (vdW) forces between the graphene layer and water molecules, which overshadowed the changes in the substrate [27]. As the plasma etching duration increased, the substrate became thinner and more porous, amplifying the contribution of vdW forces between graphene and water. Consequently, the contact angle of the S*-PG10 membranes converged to a fixed value, close to the intrinsic contact angle of water on porous graphene (Figure 6b). These findings align with the results reported by Shih et al. [28].

Figure 6c–h summarize the thickness and mechanical properties of S* substrates optimized via oxygen plasma treatment and the corresponding S*-PG10 composite FO membranes. The thickness of the S* substrates decreased with increasing plasma etching time due to oxidation by plasma-generated free radicals, which introduced defects in the substrate structure. Simultaneously, the porosity of the substrates increased significantly, from 81.8% (S) to 89.1% (S*30), which facilitated reduced mass transfer resistance for water molecules within the substrate (Figure 6d) [29]. However, the enhanced porosity came at the expense of mechanical robustness. As the plasma etching duration increased, the mechanical properties of the S* substrates deteriorated significantly. For the unmodified S substrate, the tensile strength, elongation at break, Young’s modulus, and toughness were 2.23 MPa, 3.5%, 88.35 MPa, and 0.094 MJ/m^3^, respectively. After 30 min of plasma etching, these values dropped to 1.08 MPa, 3.0%, 52.33 MPa, and 0.040 MJ/m^3^ (Figure 6e–h).

A similar trend was observed for the composite S*-PG10 FO membranes. The mechanical properties (i.e., tensile strength, elongation at break, Young’s modulus, and toughness) declined significantly as the plasma etching duration increased. This deterioration is attributed to the introduction of defects and increased porosity in the substrate, which undermined the structural integrity of both the substrate and the composite FO membranes. Unlike the findings in Figure 3c–f, where the graphene layer notably enhanced the mechanical strength of the composite FO membrane (S-PG), the porous graphene layer had a negligible effect on the mechanical strength of S*-PG10.

#### 3.2.3. FO Performances of S*-PG10 Membranes

**Figure 7:**
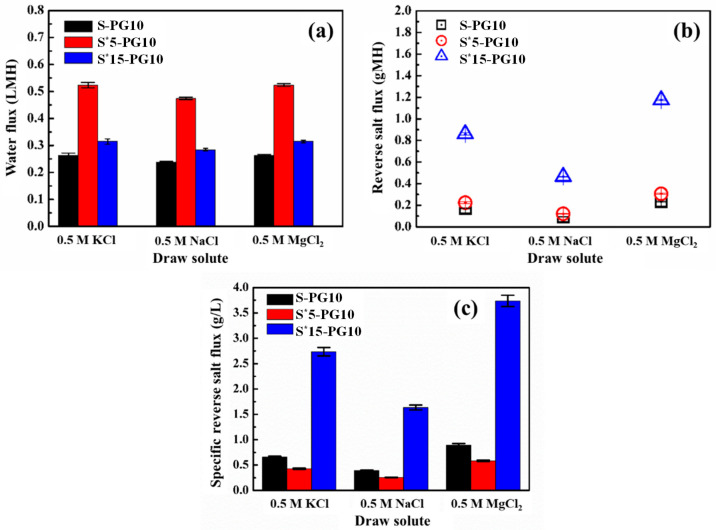
Water fluxes (**a**), RFSs (**b**) and the specific reverse salt flux (**c**) of the S*-PG10 membranes in FO using 0.5 M KCl, 0.5 M NaCl and 0.5 M MgCl_2_ as draw solutions.

FO performance of the S*-PG10 in different DSs (0.5 M KCl, NaCl, and MgCl_2_) was systematically evaluated. Given the insufficient mechanical strength of S*30-PG10 for long-term FO operation, only the FO performance of S*5-PG10 and S*15-PG10 was evaluated. Figure 7 shows that *J_w_*, *J_s_*, and *J_s_*/*J_w_* all exhibit highly consistent changes with plasma etching time in the three DSs. It indicates that the etching modification effect of the membrane is universal.

Taking 0.5 M NaCl as an example, the *J_w_* of S*5-PG10 after 5 min of etching increases to 0.47 LMH, which is 100% higher than that of the unetched S-PG10 (0.24 LMH). However, when the etching time is extended to 15 min (S*15-PG10), the *J_w_* does not continue to increase but instead drops back to 0.28 LMH. In terms of selectivity, the trend of *J_s_* is more pronounced: the *J_s_* of the S-PG10, S*5-PG10 and S*15-PG10 is 0.09, 0.12 and 0.47 gMH respectively. This difference is directly reflected in the salt water flux ratio that characterizes the overall membrane efficiency: the *J_s_*/*J_w_* of S*5-PG10 is the lowest (0.26), while that of S*15-PG10 is significantly increased to 1.64. These data clearly indicate that moderate plasma etching (5 min) is the key point for achieving the synergistic optimization of *J_w_* and *J_s_*; excessive etching leads to the impairment of the separation layer’s sieving function and a sharp increase in salt leakage. This nonlinearity is the key finding.

To explain the reasons for the above performance changes, we analyzed the structural parameters of S* substrates. The calculation results of the mass transfer resistance index (*t*/*ε*, a simplified representation of the S value) (Table 1) show that as the etching time increased from 0 s to 15 min, the *t*/*ε* values of S* substrates continuously decreased from 75.8 μm to 59.8 μm. However, the optimal FO performance of S*-PG10 does not occur at the lowest *t*/*ε* value but rather at its moderate 67.8 μm (S*5-PG10). This indicates that moderate etching can effectively alleviate ICP to enhance *J_w_* while maintaining the complete sieving function of the graphene separation layer to control salt leakage; excessive etching, however, destroys the pore structure of graphene, resulting in the loss of sieving function and thereby causing salt leakage. Therefore, the final performance is determined by the synergistic effect of optimizing the mass transfer of the supporting layer and maintaining the integrity of the separation layer structure.

### 3.3. Characterizations and Forward Osmosis Performances of S^#^-PG Membranes

As discussed earlier, while oxygen plasma treatment enhances the hydrophilicity of the Psf substrate and improves the FO performance of the PG composite membrane, it also weakens the membrane’s mechanical properties, posing challenges for stability during long-term FO operation. To address this and further strengthen the mechanical properties of PG membranes after plasma treatment, PDLLA was introduced as a doping material. It was blended with Psf to prepare the substrates (S^#^), thereby reinforcing the mechanical properties of the resulting PG composite membrane (S^#^-PG). Then, the S^#^ substrates and S^#^-PG membranes were systematically evaluated.

#### 3.3.1. Physiochemical Properties of S^#^ Substrates and the Composite S^#^-PG10 Membranes

The S^#^ substrates were prepared by blending PDLLA with Psf at different mass ratios, while the optimal etching duration of 10 s for the graphene layer and substrate etching duration of 5 min were maintained, thereby reinforcing the mechanical properties of the resulting PG composite membrane (S^#^-PG10). The surface morphology, wettability, mechanical properties, and physiochemical characteristics of the S^#^ substrates, along with the hydrophilicity of their corresponding S^#^-PG10 membranes, were systematically analyzed.

**Figure 8:**
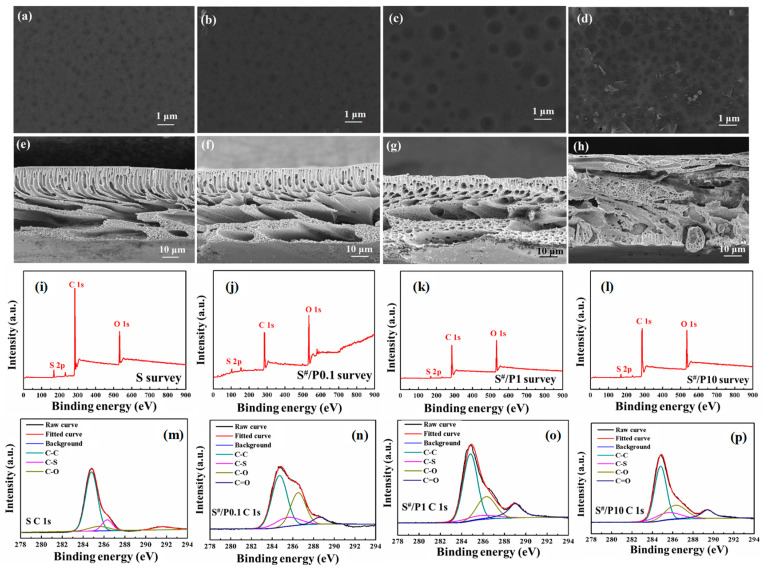
SEM images of the upper surfaces (**a**–**d**) and cross sections (**e**–**h**) of S^#^ substrates doped with different PDLLA content; XPS survey spectra (**i**–**l**) and the C 1s XPS spectra (**m**–**p**) of S^#^ substrates.

The SEM results (Figure 8a–h) show that increasing PDLLA doping from 0.1 wt.% to 10 wt.% enlarges surface pore size, reduces finger-like pore layer thickness, and ultimately transitions the support layer to a crystalline structure at 10 wt.%. Table S6 demonstrates that increasing PDLLA content enhances support layer hydrophobicity (contact angle rising from 67.5° to 90°) due to hydrophobic groups and surface roughness, while PWP value initially improves (0.1–1 wt.% PDLLA) with larger pores and higher porosity but declines at 10 wt.% PDLLA as internal densification offsets surface macropore benefits, reducing permeability. In terms of the mechanical properties (Table S7), PDLLA doping improves the support layer’s strength without affecting thickness compared to the ones with no PDLLA addition; however, at 10 wt.% PDLLA, tensile strength, elongation at break, and Young’s modulus decline as PDLLA enhances the blended membrane’s toughness. XPS and FTIR (Figure 8i–p and Figure S3) analyses confirmed that increasing PDLLA doping reduces polysulfone-derived sulfur (S) content and introduces ester-linked C=O bonds, while higher PDLLA concentrations strengthen C=O signals and weaken polysulfone-specific peaks, verifying PDLLA integration and compositional shifts in the blended support layers. Furthermore, the contact angle measurement results in Table S8 reveal that both the S-PG and S^#^-PG series membranes exhibit comparable hydrophilicity, with angles near 45°, indicating minimal impact of PDLLA incorporation on FO membrane surface wettability. These structural modifications contributed to the improved mechanical robustness and altered water transport behaviors of the S^#^-PG FO composite membranes.

#### 3.3.2. FO Performances of S^#^-PG10 Membranes

**Figure 9:**
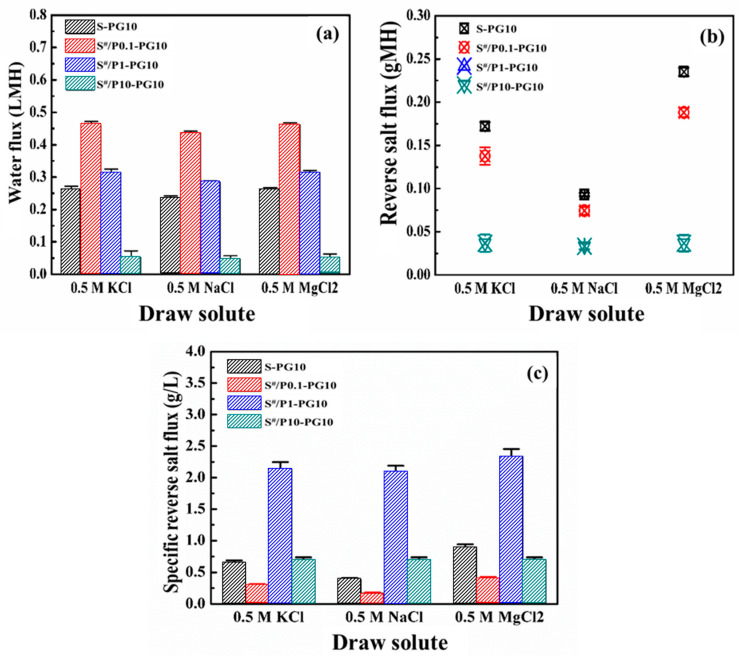
Water fluxes (**a**), RFSs (**b**) and the specific reverse salt flux (**c**) of S-PG10 and S^#^-PG10 membranes in FO using 0.5 M KCl, 0.5 M NaCl and 0.5 M MgCl_2_ as draw solutions.

Figure 9 compares the *J_w_*, *J_s_*, and *J_s_*/*J_w_* of the S-PG10 membrane and S^#^-PG10 series membranes using 0.5 M KCl, NaCl, and MgCl_2_ as DSs. As shown in Figure 9a, S^#^-PG10 membranes with 0.1~1 wt.% PDLLA doping exhibited higher *J_w_* than the S-PG10 membrane across all DSs. However, increasing PDLLA to 10 wt.% (S^#^/P10-PG10) significantly reduced *J_w_*, attributed to heightened substrate hydrophobicity (Table S6) and the formation of a crystalline structure with elevated hydraulic resistance (Figure 8a–h). Notably, while S^#^/P0.1-PG10 achieved the highest *J_w_* within the S^#^-PG10 series, its fluxes remained lower than non-PDLLA counterparts (e.g., S*5-PG10 in Figure 7a), likely due to PDLLA-induced hydrophobicity reducing water permeation.

The S^#^-PG10 series consistently demonstrated lower RSF than both the S-PG10 and S*-PG10 membranes (Figure 7b and Figure 9b), suggesting that PDLLA-modified pore structures and reduced substrate hydrophilicity hinder salt transport. This aligns with prior studies linking hydrophilic substrates to enhanced solute permeation [20]. Reduced hydrophilicity in S^#^-PG10 membranes thus effectively suppressed salt diffusion.

Specific RSF followed the order S^#^/P1-PG10 > S^#^/P10-PG10 > S-PG10 > S^#^/P0.1-PG10 (Figure 9c). This trend highlights that minimal PDLLA doping (0.1 wt.%) optimizes selectivity by reducing *J_s_* without significantly compromising *J_w_*, outperforming both undoped and higher PDLLA-doped membranes. These findings demonstrate that optimal PDLLA incorporation in Psf substrates enhances mechanical robustness while preserving favorable FO performance, achieving a critical balance between structural integrity and separation efficiency. Furthermore, a benchmarking comparison with representative graphene-based FO membranes (Table S9) reveals that our membrane achieves an exceptionally low reverse salt flux *J_s_* of 0.12 gMH. Despite the relatively lower absolute water flux due to the monolayer configuration, the competitive *J_s_*/*J_w_* ratio underscores the membrane’s potential for high-purity osmotic separation applications. While the membrane demonstrated stable performance over a 24 h evaluation period, further investigation into long-term cyclic operation and fouling resistance is required to fully assess its industrial viability.

## 4. Conclusions

This study systematically investigated the design and optimization of oxygen plasma-modified graphene composite membranes for enhanced forward osmosis (FO) performance, focusing on balancing water permeability, salt rejection, and mechanical robustness. The key findings are as follows:

(i) Oxygen plasma treatment introduced nanopores and oxygen-containing groups into monolayer graphene, enhancing its hydrophilicity and increasing the water flux by one order of magnitude compared to the S-G membrane, while maintaining over 96% salt rejection. Prolonged etching (>20 s) raised reverse salt flux (RSF) due to excessive defects, highlighting the need to optimize etching duration.

(ii) Short-term oxygen plasma treatment (5 min) of Psf substrates enhanced hydrophilicity and porosity, doubling the water flux of S*-PG10 membranes. Extended etching (>15 min) weakened mechanical strength and increased RSF by disrupting pore integrity.

(iii) PDLLA incorporation into Psf substrates (0.1~1 wt.%) improved mechanical robustness without adding thickness. The S^#^/P0.1-PG10 membrane showed optimal FO performance with reduced RSF and sustained water flux, while 10 wt.% PDLLA induced hydrophobicity and crystalline structures, lowering permeability.

Overall, this study reveals that pore architecture, surface chemistry, and substrate hydrophilicity critically govern FO efficiency. Plasma etching and PDLLA doping synergistically optimize these factors, with short-term plasma treatment and low PDLLA content striking the best balance between water flux, RSF, and mechanical durability. Future efforts to improve the FO process should focus on narrowing the nanopore size distribution via pulsed plasma etching, engineering gradient-porosity supports to further mitigate ICP, and integrating the FO with renewable-energy-driven draw regeneration (such as solar energy) for sustainable desalination.

## Figures and Tables

**Table 1:** The structure parameters of S*-PG10 membranes.

Membrane	S-PG10	S*5-PG10	S*15-PG10
*t*/*ε* (μm)	75.8	67.8	59.8

## Data Availability

The datasets presented in this article are not readily available as the data are part of an ongoing patent application. Any inquiries can be directed to the corresponding authors.

## Supplementary Materials

The following supporting information can be downloaded at: https://www.mdpi.com/article/10.3390/membranes16030104/s1, Table S1: Component ratios of the Psf/PDLLA membrane casting solution; Table S2: Preparation conditions of the S-PG composite membranes by oxygen plasma method; Table S3: Preparation conditions of the S*-PG composite membranes by oxygen plasma method; Table S4: Draw solute rejection (R) of S and S-PG membranes over a 24-h of FO process; Table S5: The surface element composition and content of the S* substrate optimized by oxygen plasma; Table S6: The static contact angles (°) and pure water permeations (PWP) of the substrates doped with different PDLLA contents; Table S7: The thickness, porosity and mechanical strengths of the substrates doped with different PDLLA contents; Table S8: The static contact angles (°) of S-PG10 and S^#^/P-PG10 series membranes; Table S9: Performance comparison of representative recent graphene FO membranes with the present work [30,31,32,33]; Figure S1: Schematic diagram of the lab-scale FO setup; Figure S2: FTIR spectra of S* substrates; Figure S3: FTIR spectra of S^#^ substrates.
