# Supplementary material for: Oxygen Plasma-Modified Graphene Composite Membranes for Enhanced Forward Osmosis Performance: Mitigating Reverse Salt Flux and Improving Permeability
Source: Membranes (Basel). 2026 Mar 16;16(3):104. doi: 10.3390/membranes16030104 (PMC13028585; doi:10.3390/membranes16030104)
Supplement: Supplementary file 1 [file membranes-16-00104-s001.zip › membranes-4155068-supplementary.pdf]

Supplementary Information

Oxygen plasma-modified graphene composite membranes for enhanced forward osmosis performance: Mitigating reverse salt flux and improving permeability

Keyuan Zhang [1,2,3,†], Yan Wu [1,2,†], Yue Jiang [1,2], Qi Han [1,2,*], Minmin Zhang [4,*], Li Feng [1,2] and Liqiu Zhang [1,2,*]

[1] Beijing Key Laboratory for Source Control Technology ofWater Pollution, College of Environmental Science and Engineering, Beijing Forestry University, 35 Qinghua East Road, Haidian District, Beijing 100083, China

[2] Engineering Research Center for Water Pollution Source Control & Ecoremediation, College of Environmental Science and Engineering, Beijing Forestry University, 35 Qinghua East Road, Haidian District, Beijing 100083, China

[3] The Institute of Seawater Desalination and Multipurpose Utilization, Ministry of Natural Resources of the People's Republic of China, Tianjin 300192, China

[4] Zhejiang Key Laboratory of Petrochemical Environmental Pollution Control, Zhejiang Ocean University, Zhoushan 316022, China

* Correspondence: hanqi@bjfu.edu.cn (Q.H.); zhangmm@zjou.edu.cn (M.Z.); zhangliqiu@bjfu.edu.cn (L.Z.)

† These authors contributed equally to this work.

Number of Tables: 9;

Number of Figures: 3.

CONTENTS:

Table S1 Component ratios of the Psf/PDLLA membrane casting solution.

Table S2 Preparation conditions of the S-PG composite membranes by oxygen plasma method.

Table S3 Preparation conditions of the S*-PG composite membranes by oxygen plasma method.

Table S4 Draw solute rejection (R) of S and S-PG membranes over a 24-h of FO process.

Table S5 The surface element composition and content of the S* substrate optimized by oxygen plasma.

Table S6 The static contact angles (°) and pure water permeations (PWP) of the substrates doped with different PDLLA contents.

Table S7 The thickness, porosity and mechanical strengths of the substrates doped with different PDLLA contents.

Table S8 The static contact angles (°) of S-PG10 and S#/P-PG10 series membranes.

Table S9 Performance comparison of representative recent graphene FO membranes with the present work.

Fig. S1. Schematic diagram of the lab-scale FO setup.

Fig. S2 FTIR spectra of S* substrates.

Fig. S3 FTIR spectra of S# substrates.

Table S1 Component ratios of the Psf/PDLLA membrane casting solution.

Substrate	Component ratios (wt.%)			
	Psf	PDLLA	PEG-400	NMP/DMF (mass ratio 3:1)
S	12	0	6	82
$S^{\#}$/P0.1	12	0.1	6	81.9
$S^{\#}$/P1	12	1	6	81
$S^{\#}$/P10	12	10	6	72

Table S2 Preparation conditions of the S-PG composite membranes by oxygen plasma method.

Membrane	Etching duration	Component composition
S	——	Psf substrate
S-G	——	Psf substrate-graphene
S-PG10	10 s	Psf substrate-monolayer porous graphene
S-PG20	20 s	Psf substrate- monolayer porous graphene
S-PG30	30 s	Psf substrate- monolayer porous graphene

Table S3 Preparation conditions of the S^{*}-PG composite membranes by oxygen plasma method.

Membrane	Etching duration	Component composition
S-PG	——	Psf substrate- monolayer porous graphene
S^{*}5-PG	5 min	modified Psf substrate- monolayer porous graphene
S^{*}15-PG	15 min	modified Psf substrate- monolayer porous graphene
S^{*}30-PG	30 min	modified Psf substrate- monolayer porous graphene

Table S4 Draw solute rejection (R) of S and S-PG membranes over a 24-h of FO process.

Membrane	Draw solute rejection (R %)		
	0.5M KCl	0.5M NaCl	0.5M $MgCl_2$
Substrate	13	17	4
S-PG10	99.995	99.999	99.999
S-PG20	99.356	99.179	99.867
S-PG30	96.677	95.766	99.346

Table S5 The surface element composition and content of the S^* substrate optimized by oxygen plasma.

substrate	Element content (%)			O/C
	S	C	O	
S	3.65	84.04	12.31	0.15
S^*5	3.80	70.95	25.25	0.36
S^*15	3.01	71.05	24.44	0.34
S^*30	2.60	76.61	20.79	0.27

Table S6 The static contact angles (°) and pure water permeations (PWP) of the substrates doped with different PDLLA contents.

Parameter	Substrate			
	S	$S^\#$/P0.1	$S^\#$/P1	$S^\#$/P10
Contact angle (°)	67.5±3.1	73.5±2.5	84±2.7	90±3.0
PWP (LMHbar)	3.47±1.1	4.26±1.3	4.6±1.9	2.1±2.7

Table S7 The thickness, porosity and mechanical strengths of the substrates doped with different PDLLA contents.

Substrate	Thickness (μm)	Porosity (%)	Tensile strength (MPa)	Elongation at break (%)	Young's modulus (MPa)	Toughness（MJ/m^3）
S	62.0±2.0	81.8	2.23	3.5	88.35	0.094
$S^\#$/P0.1	61.0±1.5	85.6	2.09	3.8	89.97	0.099
$S^\#$/P1	61.8±1.8	86.3	2.33	3.9	90.24	0.126
$S^\#$/P10	62.1±0.9	78.7	1.89	3.0	52.33	0.348

Table S8 The static contact angles (°) of S-PG10 and $S^\#$/P-PG10 series membranes.

Membrane	Contact angle (°)
S-PG10	42.5±2.0
$S^\#$/P0.1-PG10	43.0±1.5
$S^\#$/P1-PG10	45.2±1.8
$S^\#$/P10-PG10	42.1±2.1

Table S9 Performance comparison of representative recent graphene FO membranes with the present work.

Membrane	Draw solution	J_w (LMH)	J_s (g/m²/h)	J_s/J_w (g /L)	Reference
PA/GO/Psf	2 M NaCl	~11	~3.498	~0.318	[1]
PA/GO-modified PES substrate	1 M NaCl	5.12	4.95	0.97	[2]
GO/LDH/GO	1 M sucrose	2.1	3.4	1.61	[3]
PA/PEG-g-GO-Psf	2 M NaCl	11.25	3.46	0.31	[4]
Monolayer PG/ PDLLA-modified PSf	0.5 M KCl	0.47	0.12	0.26	This work

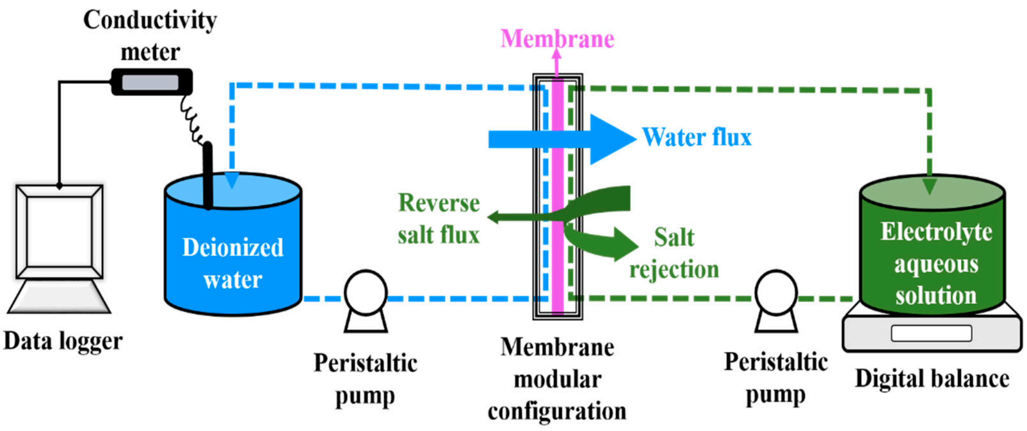

Figure S1 Schematic diagram of the lab-scale FO setup.

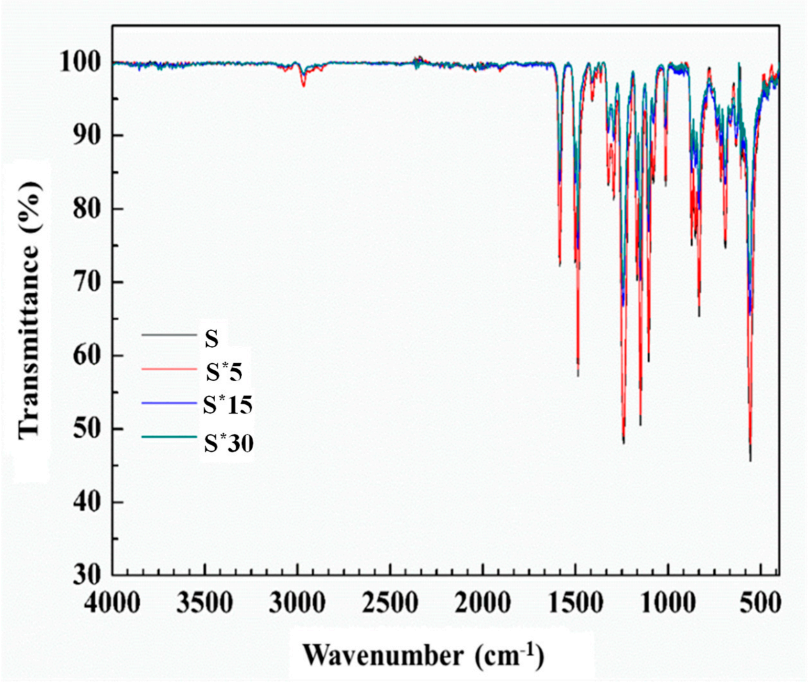

Figure S2 FTIR spectra of S^* substrates.

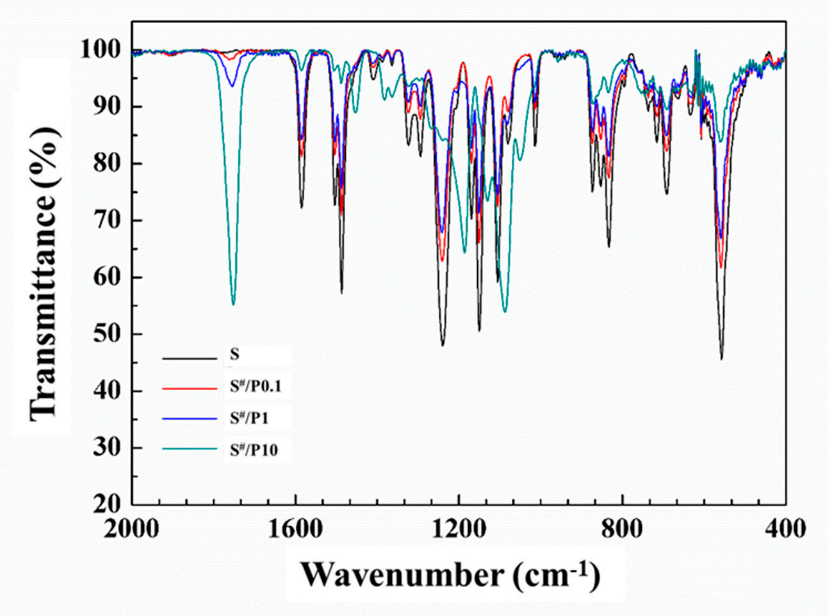

Figure S3 FTIR spectra of $S^{\#}$ substrates.

[1] T. Sirinupong, W. Youravong, D. Tirawat, W.J. Lau, G.S. Lai, A.F. Ismail, Synthesis and characterization of thin film composite membranes made of PSF-TiO2/GO nanocomposite substrate for forward osmosis applications, Arabian Journal of Chemistry, 11.

[2] H.E. Almansouri, M. Edokali, M.N.A. Seman, E.P.N. Ntone, C.K.M.F.C.K. Yahya, A.W. Mohammad, Enhanced Desalination Performance of Thin-Film Composite Forward Osmosis Membranes Through Multilayer Graphene Oxide-Modified Mixed-Matrix Polyethersulfone Substrates, Arabian Journal for Science and Engineering, (2025).

[3] L. Wang, Z. Yuan, Y. Zhang, W. Guo, X. Sun, X. Duan, Sandwich layered double hydroxides with graphene oxide for enhanced water desalination, Science China Materials, 65 (2022) 803-810.

[4] T. Ghanbari, A. Sharif, M. Karimi, Polysulfone substrates modified with polyethylene glycol-grafted graphene oxide nanosheets for enhanced forward osmosis performance, Chemical Engineering Research and Design, 217 (2025) 223-234.
